# Fluorine-18 Labelled Radioligands for PET Imaging of Cyclooxygenase-2

**DOI:** 10.3390/molecules27123722

**Published:** 2022-06-09

**Authors:** Jatinder Kaur, Atul Bhardwaj, Frank Wuest

**Affiliations:** 1 Department of Oncology, University of Alberta, Edmonton, AB T6G 1Z2, Canada; abhardwa@ualberta.ca; 2 Faculty of Pharmacy and Pharmaceutical Sciences, University of Alberta, Edmonton, AB T6G 1Z2, Canada; 3 Department of Chemistry, University of Alberta, Edmonton, AB T6G 1Z2, Canada

**Keywords:** COX-2, inhibitor, radiotracer, fluorine-18, radionuclide, radiolabelling, radioligands, PET imaging and biomarker

## Abstract

Molecular imaging probes enable the early and accurate detection of disease-specific biomarkers and facilitate personalized treatment of many chronic diseases, including cancer. Among current clinically used functional imaging modalities, positron emission tomography (PET) plays a significant role in cancer detection and in monitoring the response to therapeutic interventions. Several preclinical and clinical studies have demonstrated the crucial involvement of cyclooxygenase-2 (COX-2) isozyme in cancer development and progression, making COX-2 a promising cancer biomarker. A variety of COX-2-targeting PET radioligands has been developed based on anti-inflammatory drugs and selective COX-2 inhibitors. However, many of those suffer from non-specific binding and insufficient metabolic stability. This article highlights examples of COX-2-targeting PET radioligands labelled with the short-lived positron emitter ^18^F, including radiosynthesis and PET imaging studies published in the last decade (2012–2021).

## 1. Introduction

Cyclooxygenase (COX) enzymes play a pivotal role in the metabolism of arachidonic acid and regulate the biosynthesis of various prostanoids under normal and pathological conditions [1,2,3,4,5,6]. COX is known to exist in two main isoforms: COX-1, COX-2. Both isoforms have similar structures and show the same cyclooxygenase and peroxidase activity to produce prostaglandin H_2_ (PGH_2_), an important precursor for the biosynthesis of other prostaglandins, prostacyclin and thromboxanes. However, COX-1 and COX-2 induction, regulation, expression, and localization sites are different [7,8,9]. 

**Figure 1:**
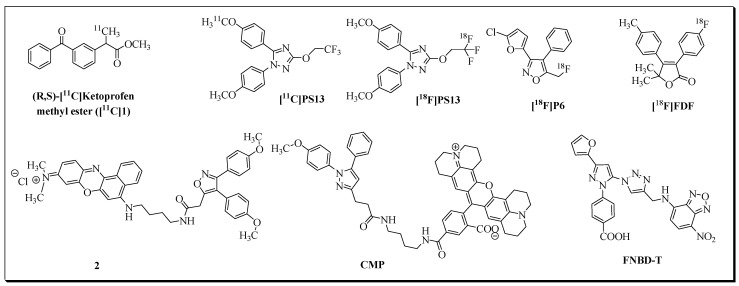
PET and fluorescent imaging probes for COX-1 isozyme.

COX-1 isozyme is encoded by the *ptgs1* gene. It is regarded as a housekeeping enzyme due to its constitutive expression in most tissues. COX-1 is involved in the biosynthesis of prostanoids required for the regulation of epithelial cytoprotection and other homeostatic cell and tissue functions [4,10]. Upregulation of COX-1 is reported in several cancers [11,12,13,14] and brain disorders associated with neuroinflammation [15,16,17,18,19]. High levels of COX-1 are observed in ovarian [12,20], prostate [21], breast [22], and cervical [23] cancer. Therefore, in addition to non-selective classical non-steroidal anti-inflammatory drugs (NSAIDs: acetylsalicylic acid, ibuprofen, flurbiprofen, naproxen, indomethacin, diclofenac, mefenamic acid, piroxicam, etc.), recently, COX-1-selective inhibitors [11,24], fluorescence imaging probes [25,26,27,28] and PET radioligands [25,26,29,30,31,32] introduced COX-1 as a promising biomarker for imaging and treatment of cancers and neuroinflammatory diseases. Selected examples of COX-1 targeting fluorescent and PET imaging probes are shown in Figure 1. Recently, to follow up the development of COX-1 targeted radioligand ([^11^C]PS13), Taddei et al. synthesized an ^18^F-labeled version of PS13, [^18^F]PS13, by following two labeling approaches based on the nucleophilic addition of [^18^F]fluoride ion to *gem*-difluorovinyl precursors [33]. Despite low molar activity, the [^18^F]PS13 radioligand was evaluated for COX-1 PET imaging in monkeys. 

**Figure 2:**
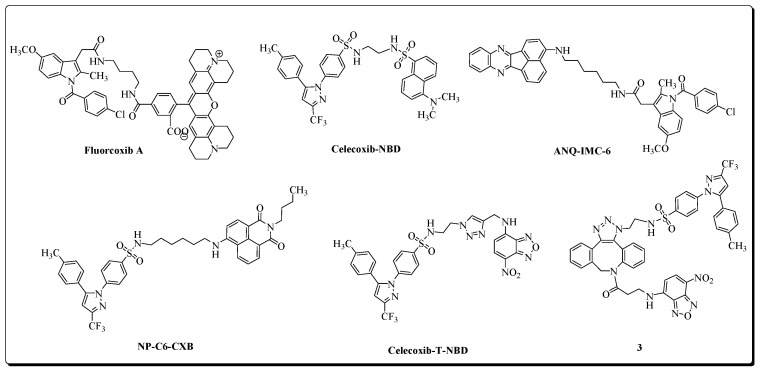
Selected examples of fluorescent probes for COX-2 imaging.

In contrast to the constitutively expressed COX-1 isozyme, the COX-2 isoform is primarily an inducible enzyme. The expression of COX-2 is induced by numerous stimuli such as proinflammatory cytokines, lipopolysaccharides, growth factors (fibroblast growth factor, platelet-derived growth factor, epidermal growth factor), hormones, and oncogenes, resulting in the production of prostaglandins (PGs) in inflamed tissues [4,34]. COX-2 is involved in several inflammatory diseases, types of cancer [35,36,37,38,39,40,41,42,43,44,45], and neurodegenerative pathways [45,46,47,48]. COX-2 selective inhibitors (Coxibs) were introduced to attenuate the gastrointestinal toxicity-related side effects of non-selective NSAIDs. COX-2 plays a crucial role in tumorigenicity, angiogenesis, metastasis, and apoptosis-resistance in cancer, including colorectal, pancreatic, brain, and breast cancer. Moreover, COX-2 is frequently upregulated in cancer, making COX-2 a promising biomarker for cancer diagnosis and therapy, as demonstrated by the development of various fluorescent [49,50,51,52,53] and radioimaging probes over the last decades [54,55,56,57]. COX-2-targeting fluorescent probes mainly consist of a fluorescent group attached to known anti-inflammatory drugs. Selected examples of COX-2 fluorescent imaging probes are presented in Figure 2. 

**Figure 3:**
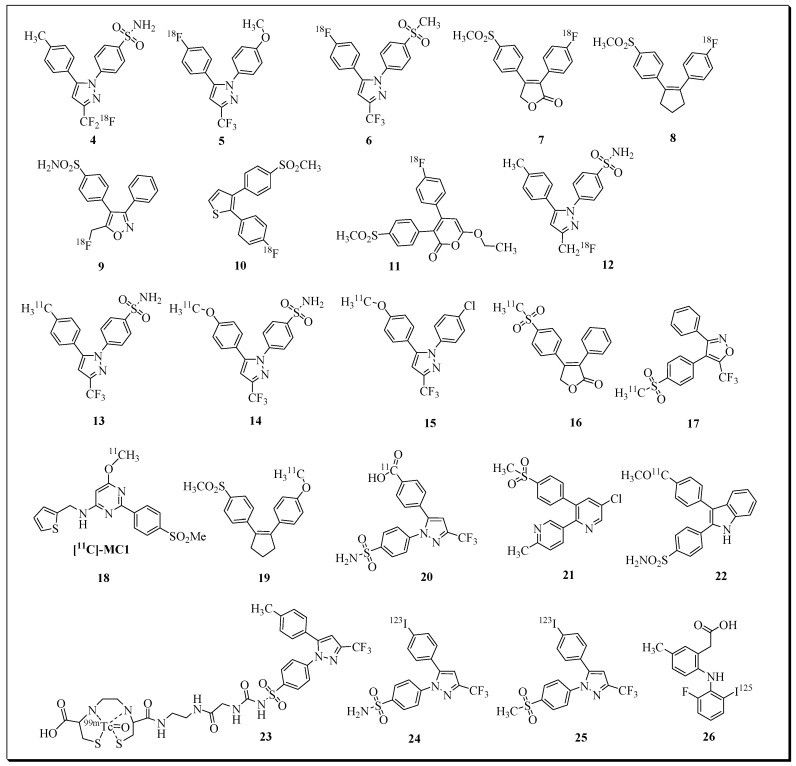
Selected examples of radioligands for COX-2 imaging.

There has also been significant progress in the development of COX-2-targeting radioligands for imaging inflammation, cancer, and neurological disorders [54,55,56,57]. Over the last decades, a variety of radionuclide-based imaging agents have been developed by the incorporation of radioisotopes such as ^11^C, ^18^F, ^99m^Tc, ^123^I, and ^125^I into NSAIDs and related compounds [55,56,58,59,60,61,62,63,64,65,66,67,68,69,70,71]. Selected examples of PET radioligands for COX-2 imaging are presented in Figure 3

A major focus involves ^18^F-labeled radioligands, due to their favourable half-life (t_1/2_ = 109.8 min), ease of production, the availability of a variety of radiofluorination methods, and better imaging characteristics of the short-lived positron emitter ^18^F. 

This review article primarily covers ^18^F-labeled radioligands reported in the last decade for targeting COX-2. A particular emphasis is on reporting radiochemistry and PET imaging data.

## 2. 2012

**Figure 4:**
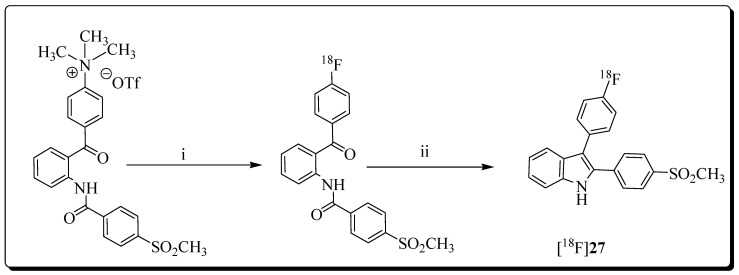
Radiosynthesis of [^18^F]**27** via McMurry coupling. Reagents: (i) [^18^F]fluoride, K_222_/K_2_CO_3_, DMF; (ii) TiCl_4_, Zn, THF.

The development of a series of 3-diarylsubstituted indole-based inhibitors was reported by Hu et al. [72]. The highly potent and selective COX-2 inhibitor 3-(4-fluorophenyl)-2-(4-methylsulfonyl-phenyl)-1*H*-indole (K_i_(COX-2) = 20 nM; K_i_(COX-1) >10 µM) was selected for the development of a novel COX-2 radioligand for PET [73]. In 2012, Kniess et al. described an innovative procedure for the radiosynthesis of 3-(4-[^18^F]fluorophenyl)-2-(4-methylsulfonylphenyl)-1*H*-indole [^18^F]-4 (Figure 4). 

The radiosynthesis involved the direct radiofluorination of a non-cyclized trimethylamino triflate precursor using [^18^F]KF and Kryptofix (K_222_). The final compound was obtained via McMurry cyclization chemistry using zinc and titanium tetrachloride in an automated radiosynthesis process, including HPLC purification and solid-phase extraction. The radiosynthesis of [^18^F]**27** was accomplished within 80 min in 10% total decay-corrected yield starting from [^18^F]fluoride. The radiochemical purity was >98%, and the molar activity reached 74–91 GBq/mol.

Kniess et al. examined the COX-1 and COX-2 inhibition profiles of compound **27**. The in vitro binding assay results revealed that compound **27** is a moderately potent and selective COX-2 inhibitor (COX-2 IC_50_ = 1.2 μM, COX-1 IC_50_ = 6.6 μM, SI = 5.5).

**Figure 5:**
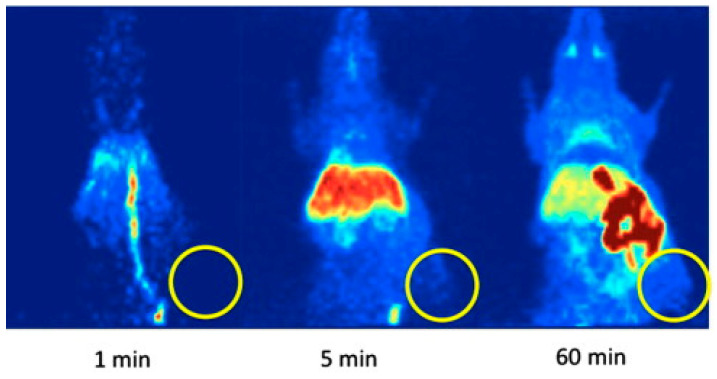
Representative PET images of a HT-29-tumor-bearing mouse (maximum intensity projections) at 1, 5, and 60 min after a single intravenous injection of [^18^F]**27** (circle: position of the subcutaneously xenotransplanted tumor). Figure readapted with permission from publisher [74].

In vivo PET imaging was performed in HT-29-tumor-bearing mice. During dynamic small animal PET studies, no substantial tumor accumulation of [^18^F]-**3** was observed (Figure 5). Most of the radioactivity was found to accumulate in the liver, small intestine, and kidney, and a small amount of activity (SUV ~ 1) was noticed in the brain and the blood pool. It was concluded that [^18^F]**27** is unsuitable for in vivo PET imaging of COX-2 in mice with xenotransplanted HT-29 tumors. 

## 3. 2013

**Figure 6:**
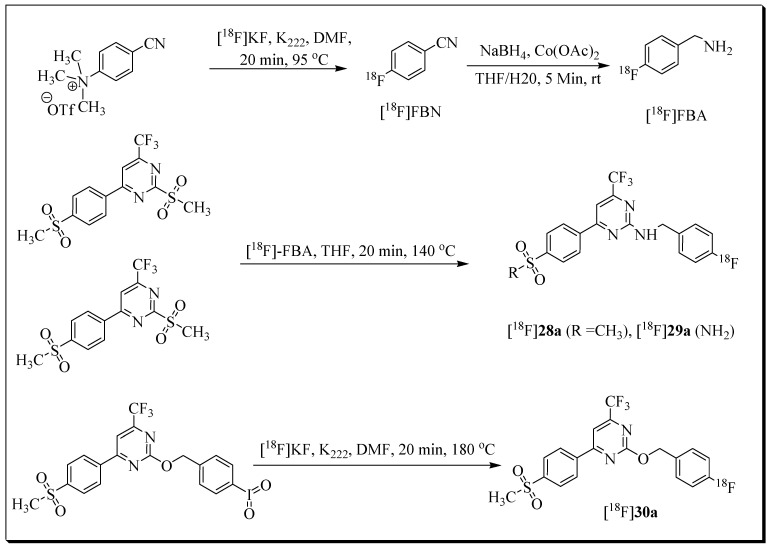
Radiosynthesis of [^18^F]**28a** [^18^F]**29a**, and [^18^F]**30a**.

In 2013, our group reported the synthesis and in vitro evaluation of a series of trifluoromethyl-substituted pyrimidines as COX-2 inhibitors. Three fluorobenzyl-substituted pyrimidine derivatives were further advanced as ^18^F-labeled radioligands ([^18^F]**28a** (^18^F-Pyricoxib), [^18^F]**29a**, and [^18^F]**30a**) [75]. Radiotracers [^18^F]**28a** and [^18^F]**29a** were synthesized using 4-[^18^F]fluorobenzylamine ([^18^F]FBA) as a building block (Figure 6). The radiosynthesis and HPLC purification of [^18^F]**28a** were accomplished within 95 min, and the decay-corrected radiochemical yield based on [^18^F]FBA was 27 ± 11%. The total synthesis time for radioligand [^18^F]**29a** was 110 min, including HPLC purification. Radioligand [^18^F]**29a** was isolated in decay-corrected radiochemical yields of 23% ± 1%. 

The radiosynthesis [^18^F]**30a** was accomplished using iodylaryl derivative as the labelling precursor, leading to a radiochemical yield of 5 to 10% (as determined by radio-TLC). 

The radiotracers were tested in COX-2-expressing human HCA-7 colorectal cancer cells, and in vitro specificity was examined using COX-2-negative HCT-116 cells, and by blocking studies with COX-2 inhibitors [76,77]

**Figure 7:**
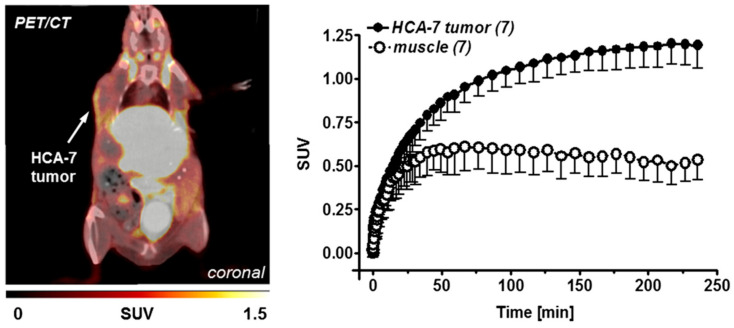
Left: PET/CT image (coronal slice) 2 h after injection of [^18^F]Pyricoxib into an HCA-7-tumor-bearing NIH-III mouse. An amount of 3.5% HSA was added as a carrier protein to the final injection solution. Right: Time-activity curves for tumor uptake of [^18^F]Pyricoxib and its clearance from muscle tissue over 4 h post injection. Data are shown as mean ± SD from seven dynamic PET experiments. Figure readapted with permission from publisher [78].

**Figure 8:**
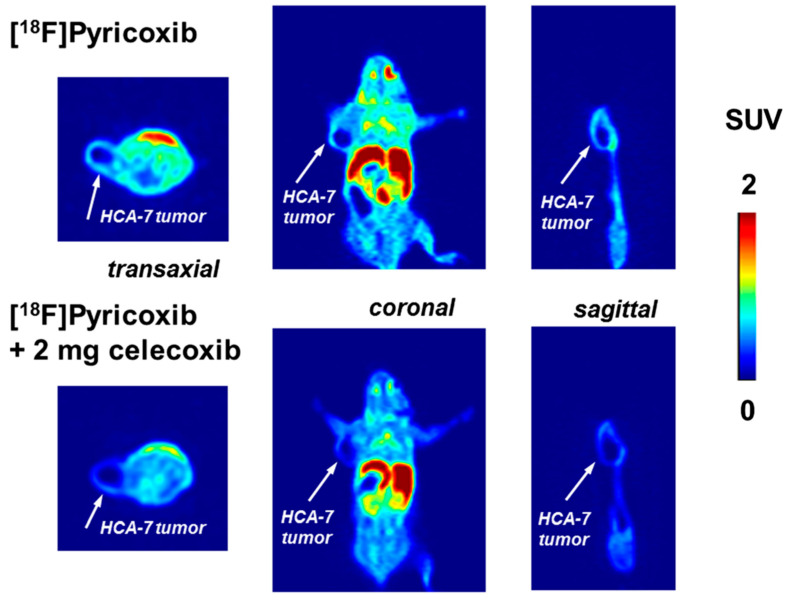
Top: transaxial, coronal, and sagittal PET images at 60 min p.i. of [^18^F]Pyricoxib into HCA-7-tumor-bearing NIH-III mouse (control); bottom: transaxial, coronal, and sagittal PET images at 60 min p.i. of [^18^F]Pyricoxib into HCA-7-tumor-bearing NIH-III mouse (pre-treated with 2 mg of celecoxib 60 min before radiotracer administration; no HSA added). Figure readapted with permission from publisher [76].

As in the cell uptake studies results in HCA-7 cells, the in vivo studies with [^18^F]**28a** (^18^F-Pyricoxib) showed uptake in HCA-7 tumors (T/M ratio of 2.25 after 4 h p.i.) (Figure 7). A reduced uptake of [^18^F]**28a** in the tumor was noticed upon pre-treatment with the COX-2 selective inhibitor celecoxib (Figure 8) [76,77]. However, a similar radioactivity uptake profile was observed for ^18^F-Pyricoxib during studies on HCT-116-tumor-bearing mice, (SUV_2h_ 0.93 ± 0.06 (HCT-116; *n* = 3) versus 1.09 ± 0.13 (HCA-7; *n* = 3). The radiopharmacological profile of [^18^F]**28a** was superior to radioligands [^18^F]**29a** and [^18^F]**30a** [77].

In 2013, two radiolabeled COX-2 selective inhibitors, [^11^C]celecoxib and [^11^C] rofecoxib, were assessed as PET tracers for imaging COX-2-normal and ischemic mouse brains [66]. From a series of experiments, including in vitro autoradiography and in vivo PET assays, the authors concluded that [^11^C]celecoxib is not a suitable COX-2 radioligand for in vitro and in vivo assays, whereas [^11^C]rofecoxib is useful for in vitro assays of COX-2. 

## 4. 2014

No ^18^F-labelled COX-2 imaging probes were reported in 2014. However, Perrone et al. reported the development of [^18^F]-**P6** for PET imaging of COX-1 expression in human OVCAR-3 (ovarian cancer) tumor xenografts (Figure 1) [79]. Radioligand [^18^F]-**P6** was prepared in 18% radiochemical yield, and PET/CT imaging analyses showed that radiotracer [^18^F]-P6 accumulated in COX-1 expressing OVCAR 3 tumors. In addition to this study, a few review articles highlighting applications of PET radioligands were published in 2014 [70,74].

## 5. 2015

**Figure 9:**
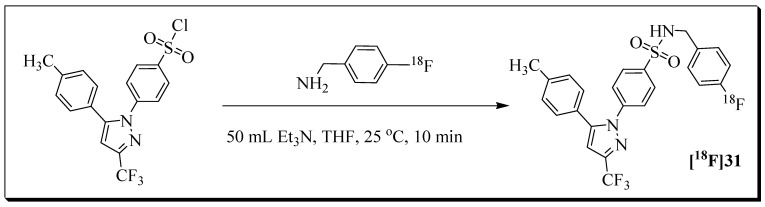
Radiosynthesis of radioligand [^18^F]**31** from labelling precursor and 4-[^18^F]fluorobenzylamine ([^18^F]FBA).

The discovery of a fluorescent probe, Celecoxib-NBD [51], in our lab led to the development of a new series of fluorine-containing cyclooxygenase-2 (COX-2) inhibitors [80]. Based on the favourable COX-2 inhibition profile, compound N-(4-fluorobenzyl)-4-(5-p-tolyl-3-trifluoromethylpyrazol- 1-yl)benzenesulfonamide **31** (IC_50_ = 0.36 µM, SI > 277) was selected for ^18^F labelling and PET imaging studies. Radioligand [^18^F]**31** was evaluated in the human colorectal cancer cell line HCA-7 (Figure 9) [81]. 

The ^18^F building block [^18^F]fluorobenzylamine ([^18^F]FBA) was used for the radiosynthesis of radioligand [^18^F]**31**. The decay-corrected radiochemical yield based on [^18^F]FBA was 20%, and the molar activity was greater than 40 GB/µmol. The total synthesis time, including HPLC purification, was 85 min.

**Figure 10:**
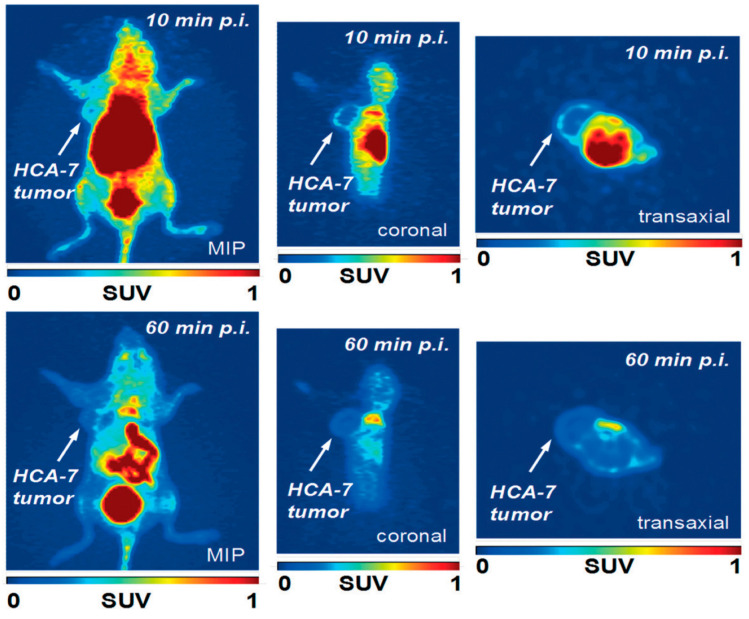
Maximum intensity projection (MIP), as well as coronal and transaxial PET images at 10 and 60 min p.i. of radioligand [^18^F]**31** in HCA-7-tumor-bearing (left flank) NIH-III mice (injected activity = 2.89 MBq) under isoflurane anesthesia. Figure readapted with permission from publisher [81].

Although the normalized cellular uptake of [^18^F]**31** in COX-2-positive HCA-7 cells was high (450% radioactivity per mg protein after 60 min), no noticeable blocking effect was observed by pre-treatment with known COX-2 inhibitors (celecoxib, rofecoxib). It was suggested that the uptake and retention of [^18^F]**31** in HCA- 7 cells is associated with unknown non-COX-2 targets. PET imaging of radioligand [^18^F]**31** in HCA-7-tumor-bearing NIH-III mice revealed an SUV_max_ in HCA-7 tumors of 0.40 ±0.07 (*n* = 3) after 10 min p.i. (Figure 10). At the same time point, the muscle uptake was 0.29 ± 0.08 (*n* = 3); therefore, the tumor-to-muscle ratio was only 1.4, providing poor imaging contrast. An analysis of the time–activity curves for radioactivity uptake in the tumor suggested that radioligand [^18^F]**31** was not trapped in tumor tissue. Therefore, radioligand [^18^F]**31** is unsuitable for COX-2 imaging. 

## 6. 2016

**Figure 11:**
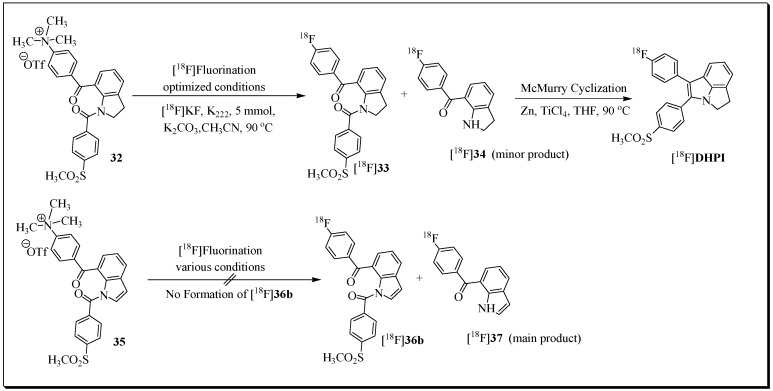
Radiosynthesis route leading to **[^18^F]DHPI**. [^18^F]Fluorination of indoline precursor **32** under optimized conditions forms intermediate [^18^F]**33**, which is subsequently cyclized to **[^18^F]DHPI** by McMurry reaction. The [^18^F]fluorination of indole precursor **35** furnished only side product [^18^F]**37** instead of [^18^F]**36,** so the McMurry reaction was not feasible.

A novel set of diaryl-substituted heterocycles containing a tricyclic dihydropyrrolo[3,2,1-hi]indole and pyrrolo[3,2,1-hi]indole core structure were designed to improve the COX-2-inhibitory activity of the previously reported compound IND [78]. From this series of compounds, two promising COX-2 selective inhibitors, **DHPI** (IC_50_ COX-2: 0.15 mM, SI = >666) and **PI** (IC_50_ COX-2: 0.04 mM, SI = >2500) were selected for ^18^F-labelling and PET imaging studies (Figure 11) [82]. Gassner et al. described the radiosynthesis of [^18^F]**DHPI** and [^18^F]**PI**. [^18^F]**DHPI** was prepared in two steps, involving the [^18^F]fluorination of trimethylammonium labelling precursor under mild conditions followed by McMurry cyclization in the presence of TiCl_4_ and zinc.

The automated synthesis afforded a decay-corrected radiochemical yield of 16% for radioligand [^18^F]**DHPI**. The total synthesis time was 110 min, and the molar activity was 45–106 GBq/mmol.

A series of cell uptake studies in five human cell lines which significantly differed in their COX-2 expression levels were conducted to confirm the COX-2-specific cell uptake of [^18^F]**DHPI**. However, blocking experiments with the COX-2 inhibitor celecoxib did not result in a significant change in the cellular uptake of [^18^F]**DHPI.** These cellular uptake study results are indicative of non-specific binding of [^18^F]**DHPI**. Additional radiopharmacological experiments were conducted in female A205-tumor-bearing NMRI nu/nu mice. Consistent with cellular uptake studies, radioligand [^18^F]**DHPI** uptake could also not be blocked in COX-2-positive A2058 tumors after pre-injection of celecoxib.

In another study, indomethacin, a known COX-1/2 inhibitor, was conjugated with zwitterionic phosphonium aryltrifluoroborates for radiolabeling with ^18^F. The radiolabeling of these novel indomethacin conjugates was achieved by innovative ^18^F-^19^F isotopic exchange chemistry [83].

**Figure 12:**
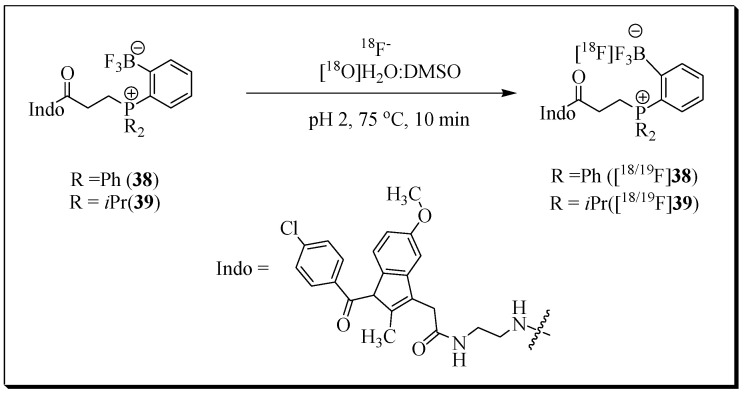
Scheme showing the radiolabeling of **38** and **39** by isotopic exchange in an aqueous solution.

Briefly, phosphonium trifluoroborate/indomethacin conjugates **38/39** were dissolved in DMSO and incubated with irradiated [^18^O]water containing ^18^F at 75 °C for 10 min. Radioligands [^18/19^F]**38** and [^18/19^F]**39** were obtained in 95.1% and 93.5% radiochemical yield, respectively (Figure 12). The study was focused on radiosynthesis, and PET imaging experiments were not reported.

## 7. 2017

Lebedev et al. utilized electrochemical radiofluorination chemistry for the radiosynthesis of ^18^F-labelled COX-2 inhibitor ^18^F-**40** (COX-2 IC_50_ = 1.5 nM) [84]. The radiolabeling occurred directly on a heteroaromatic ring. The electrochemical ^18^F-labelling was performed using a radioelectrochemical synthesizer built in-house. Briefly, radiolabeling involved ^18^F drying on a cartridge and pushing precursor through the cartridge into a Teflon reactor. The reaction mixture was electrolyzed at ambient temperature using 1.0 mm Pt wire electrodes. The electrolysis and fluorination in the cell were performed for 70 min using an Autolab PGSTAT204. The slurry was subjected to purification, followed by treatment with HCl in a pre-heated reactor for 15 min.

**Figure 13:**
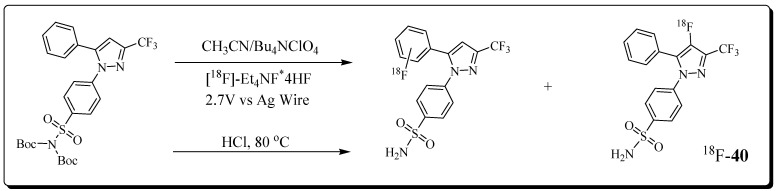
The reaction scheme of the electrochemical radiosynthesis of COX-2 inhibitor ^18^F-**40**.

The radiosynthesis of ^18^F-**40** was completed in 4 h, and the radioligand was prepared in 0.8–2% decay-corrected radiochemical yield. The molar activity was approximately 3 Ci/mmol (Figure 13).

The authors observed a COX-2-dependent cell uptake of ^18^F-**40** in LPS-treated RAW264.7 macrophage-like cells. A reduction of radioligand uptake was detected when cells were pre-treated with the selective COX-2 inhibitor celecoxib. In vivo metabolism and PET imaging studies with radioligand ^18^F-**40** in healthy mice showed no retention in bones over 2 h, which is indicative of no radiodefluorination in vivo. The radioligand crossed the blood–brain barrier and showed excretion mainly through the hepatobiliary pathway. Radioligand ^18^F-**40** also displayed rapid blood clearance and high metabolic stability in vivo. Overall, radioligand ^18^F-**40** possesses favourable characteristics suitable for PET imaging of COX-2.

**Figure 14:**
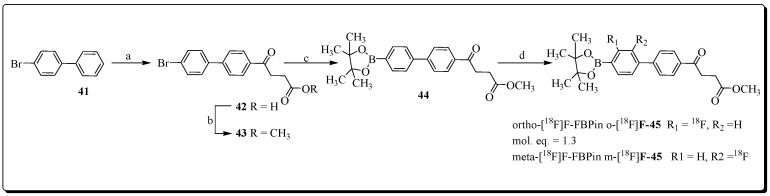
Preparation of boronofenbufen analogs O- and m-[^18^F]-**45**. Reagents and condition (a) dihydrofuran-2,5-dione, AlCl_3_; (b) CH_3_OH/H_2_SO_4_, 96% over two steps; (c) Bis (pinacolato)diboron, AcOK, Fe(C_5_H_4_)2(PPh_2_)_2_PdCl_2_, DMF, 85%; (d) CH_3_COO[^18^F]F/CF_3_COOH, 8%.

Yeh et al. reported the development of *m*-[^18^F]fluorofenbufen ester boronopinacol (*m*-[^18^F]FFBPin) for boron neutron capture therapy (BNCT) of aggressive cholangiocarcinoma (CCA) that overexpresses COX-2 enzyme [85]. Electrophilic radiofluorination chemistry of protected fenbufen boronopinacol (FBPin) **44** was accomplished using CH_3_COO[^18^F]F. This radiofluorination reaction produced two products, the desired product meta-[^18^F]fluorofenbufen pinacol (*m*-[^18^F]FFBPin) and ortho-[^18^F] fluorofenbufen pinacol (*o*-[^18^F]FFBPin), in a radiochemical yield of 8% at a molar activity of 15 MBq/μmol (Figure 14).

Radioligand *m*-[^18^F]FFBPin showed moderate COX-2 inhibition and selectivity (COX-2 IC_50_ = 0.33 ± 0.24 μM, COX-1 IC_50_ = 0.91 ± 0.68 Μm) in a competitive binding assay. The assessment of radiotracer *m*-[^18^F]FFBPin in a COX-2 overexpressing CCA tumor rat model showed a maximum tumor uptake at 10 min; afterwards, radioligand *m*-[^18^F]FFBPin was washed out from tumor tissue over 60 min. No reduction of *m*-[^18^F]FFBPin uptake levels was noticed in the presence of FBPin 4 (4 mg) during blocking studies.

During BNCT investigations, the CCA rats treated with FBPin (20–30 mg) showed a reduction in tumor size. This therapeutic outcome of FBPin is encouraging and suggests that novel boron-containing COX-2 inhibitors should be further explored for BNCT.

## 8. 2018

Elie et al. synthesized and evaluated a series of (2,3-di(het)arylated (aza)indazole-based compounds as novel COX-2 inhibitors. Compound 4-[3-(4-fluorophenyl)indazol-2-yl]benzene-sulfonamide (**49,** IC_50_ = 0.409 µM) was identified as the lead candidate for radiolabeling with ^18^F, and subsequent PET imaging in a rat model of neuroinflammation was conducted [80]. The radiosynthesis of [^18^F]**49** followed three different radiolabeling strategies. The first approach failed and encompassed direct radiofluorination chemistry starting from a nitro labelling precursor. Second, ^18^F labelling was performed using an iodonium salt precursor. However, this approach also failed and yielded only minimal amounts of radioligand [^18^F]**49**.

**Figure 15:**
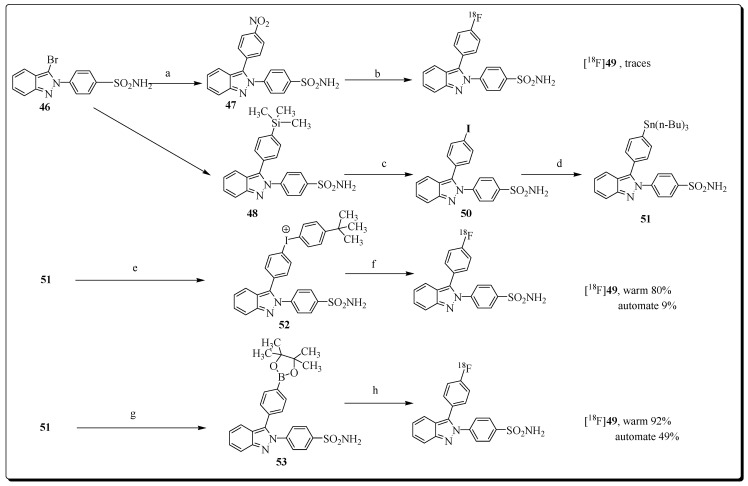
Reagents and conditions: (a) corresponding boronic acid (1.2 equiv), Cs_2_CO_3_ (3.0 equiv), Pd(PPh3)4 (0.1 equiv), dioxane, 150 °C, lW, 1 h, 42 53%, **25** 69%; (b) [K/K_222_]^+ 18^F^−^, DMF, 130 °C, lW, 20 min; (c) ICl (2.0 equiv), CH_2_Cl_2_, 0 °C then t.a., 1.5 h, 99%; (d) n-Bu_6_Sn_2_ (3.3 equiv), Pd(PPh_3_)_4_ (0.1 equiv), dioxane, 90 °C, 2 h, 33%; (e) Moser reagent (1.5 equiv), CH_3_CN/CH_2_Cl_2_ (1/1), r.t., 18 h, 73%; (f) [K/K_222_]^+ 18^F^−^, DMF, Cu(OTf)_2_ cat, 100 °C, lW, 10 min; (g) 1,4-bis(4,4,5,5-tetramethyl-1,3,2-dioxaborolan-2-yl)benzene (3.0 equiv), Na_2_CO_3_ (6.5 equiv.), Pd(PPh_3_)_4_ (0.02 equiv), dioxane, 100 °C, lW, 40 min, 52%; (h) KOTf; K_2_CO_3_, [^18^F]KF, Cu(OTf)_2_ cat., DMF, pyridine, 130 °C, 20 min.

Finally, with the use of boronic ester-based precursor **53**, the automated radiosynthesis of [^18^F]**49** was accomplished in 46% decay-corrected radiochemical yield within 20 min (Figure 15).

In vivo PET imaging of radioligand [^18^F]**49** in a rodent model of neuroinflammation showed uptake in several cerebral regions (cerebellum, striatum, cortex, hippocampus). However, no significant increase of [^18^F]**49** uptake in inflamed brain regions was noticed.

High levels of non-specific binding of [^18^F]**49** was confirmed by blocking studies with COX-2 inhibitor, as no reduced uptake of radioligand [^18^F]**49** was observed in peripheral tissues and organs. Overall, radioligand [^18^F]**49** was unsuccessful for PET imaging of COX-2 in models of neuroinflammation.

**Figure 16:**
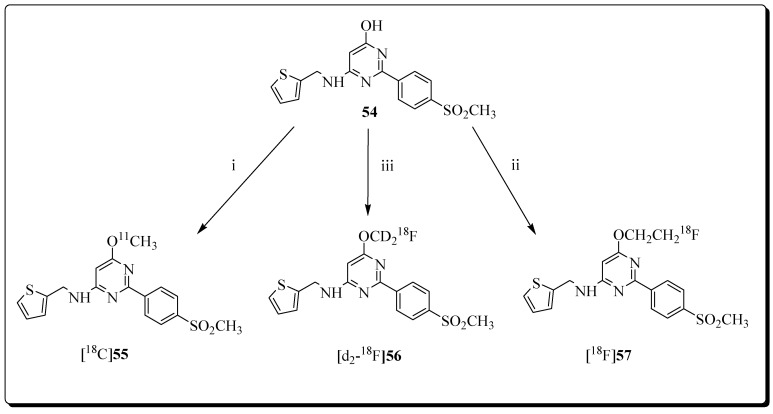
Radiosyntheses of [^11^C]**55**, [^18^F]**56**, and [^18^F]**57**. Reagents and conditions: (i) [^11^C]CH_3_I, DMF, TBAH, RT, 5 min; (ii) [^18^F]FCH_2_CH_2_Br, DMF, Cs_2_CO_3_, 18-crown-6, 110 °C, 15 min; (iii) [^18^F]FCD^2^Br, Cs_2_CO_3_, 18-crown-6, 110 °C, 15 min.

Cortes-Salva et al. described the synthesis and COX-2 inhibition analysis of a series of 2(4-methyl-sulfonylphenyl)pyrimidine-based compounds [86]. Among eleven COX-2 inhibitors, three compounds (6-methoxy-2-(4-(methylsulfonyl)phenyl-N-(thiophen-2ylmethyl)pyrimidin-4-amine (**55**), 6-fluoromethyl analogue (**57**), and 6-(2-fluoroethoxy) analogue (**56**)) were selected for radiolabeling based on their favourable COX-2-inhibitory profile, selectivity, and lipophilicity. Radioligand [^11^C]**55**, was prepared in 1.85–3.11 GBq yield upon reacting precursor **54** with [^11^C]iodomethane under basic conditions for 5 min. The molar activity was 204–492 GBq/μmol (Figure 16).

The radiofluorination of precursor **54** was performed with 2-[^18^F]fluoro-1-bromoethane, and radioligand [^18^F]**57** was obtained in 8.4% radiochemical yields within 15 min at a molar activity of 60 GBq/μmol. Labeling precursor **54** was also used for the preparation of radioligand [*d*_2_-^18^F]**56**. Compound **54** was reacted with double deuterated agent [*d*_2_-^18^F]fluorobromomethane in the presence of Cs_2_CO_3_, and 18-crown-6 at 110 °C for 15 min. Radioligand [*d*_2_-^18^F]**56** was isolated in 27% yield at a molar activity of 66 GBq/μmol. In summary, the authors identified promising PET radioligands for imaging COX-2, and established several methods for radiosynthesis. No PET imaging data using the novel radioligands have been reported.

**Figure 17:**
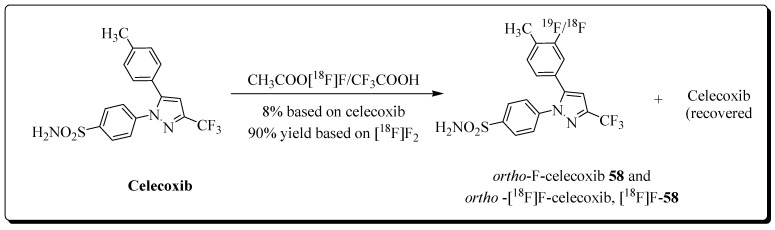
Preparation of *ortho*-[^18^F]F-**58**.

In 2018, Chang et al. reported the synthesis and evaluation of *ortho*-[^18^F]fluorocelecoxib for PET imaging of COX-2 in a cholangiocarcinoma (CAA) model [87]. Celecoxib was reacted with a CH_3_COOF/[^18^F]CH_3_COOF mixture for 25 min, and the radioligand *ortho*-[^18^F]F-**58** was obtained in 9% radiochemical yield (Figure 17). Following the COX-1/2 inhibition analyses of *ortho*-[^18^F]F-**58** (COX-2 IC_50_ = 39.0 nM, COX-1 = 24.5 mM), the radioligand was tested for COX-2 uptake in CAA cells and tumor models. PET studies demonstrated the accumulation of *ortho*-[^18^F]F-**58** in CAA tumors, although no reduction in activity levels could be observed during blocking experiments with celecoxib.

## 9. 2019

In 2019, no reports related to ^18^F based COX-2 targeting PET probes were published. However, Uddin et al. reported the development of radioligand [^18^F]FDF for PET imaging of COX-1 in an ovarian cancer model [32]. The authors described the identification of COX-1 selective inhibitor 3-(4-fluorophenyl)-5,5- dimethyl-4-(p-tolyl)furan-2(5H)-one (FDF), possessing acceptable in vivo stability, plasma half-life, and pharmacokinetic properties for its application as an imaging agent.

**Figure 18:**
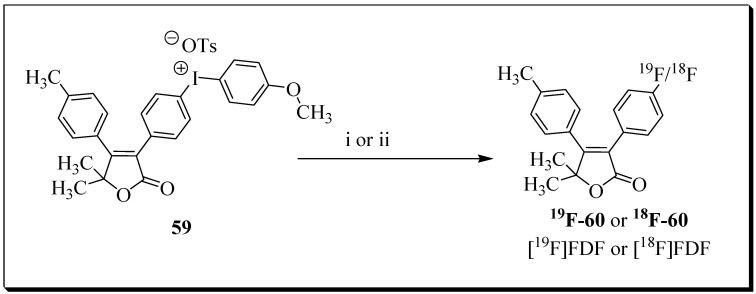
Synthesis of [^18^F]FDF: (i) [^19^F]-Bu4NF, m-CPBA, DMSO, 80 °C, 30 min; (ii) Bu_4_NHCO_3_, ^18^F-fluoride, *m*-CPBA, DMSO, 80 °C, 30 min.

[^18^F]FDF radiosynthesis was tested with several reaction conditions, and ultimately the radioligand was obtained via Lewis acid-catalyzed nucleophilic aromatic deiodo[^18^F]fluorination reaction using tetrabutylammonium ^18^F-fluoride and labelling precursor **59** (Figure 18).

Radioligand [^18^F]FDF was obtained in 7.4% decay-corrected radiochemical yield [*n* = 14 batches] at a molar activity of 578.8 Ci/mmol with 99.9% radiochemical purity. The overall synthesis time was 50 min. PET imaging studies with the radioligand [^18^F]FDF in COX-1-overexpressing ovarian cancer models in mice showed COX-1-mediated uptake in the tumors compared to normal tissues. The study presented radioligand [^18^F]FDF as a promising lead compound for further preclinical and clinical development.

## 10. 2020

**Figure 19:**
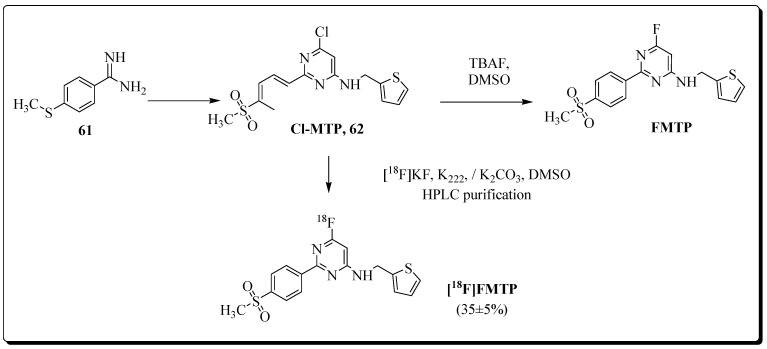
Synthesis of FMTP and radiosynthesis of [^18^F]FMTP.

Kumar et al. reported the synthesis and evaluation of pyrimidine-based radiotracer [^18^F]6-fluoro-2 (4-(methylsulfonyl)phenyl)-N-(thiophen-2-ylmethyl)pyrimidin-4-amine ([^18^F]FMTP) [88]. The radiotracer was synthesized by the replacement of chloride substituent with ^18^F on position 6 of the pyrimidine ring of labelling precursor. Radioligand [^18^F]FMTP was isolated in 35 ± 5% radiochemical yield with >99% radiochemical purity and a molar activity of 92.5 ± 18.5 GBq/μmol (*n* = 10) (Figure 19).

COX-2 binding affinity was screened by cell uptake experiments in COX-2 positive BxPC3 cells and COX-2 negative PANC1 cells. More uptake of radioligand [^18^F]FMTP was noticed in BxPC3 cells compared to PANC1 cells. FMTP was more effective during blocking experiments than celecoxib for blocking radioligand uptake in BxPC3 cells.

PET imaging studies with radioligand [^18^F]FMTP in normal mice demonstrated that the radioligand was able to cross the blood–brain barrier. However, the radiotracer also showed rapid brain wash-out within the first few minutes. An approximately two times higher uptake of radioligand [^18^F]FMTP in the brain was noticed in an LPS-induced neuroinflammation model compared with PBS-treated mice.

The preliminary studies of the radioligand showed promising first results, and additional radiopharmacological studies are needed to validate and understand the COX-2-specific uptake in the neuroinflammation model.

**Figure 20:**
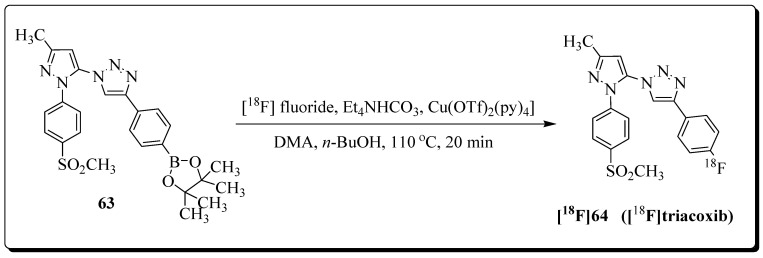
Synthesis of [^18^F]triacoxib.

**Figure 21:**
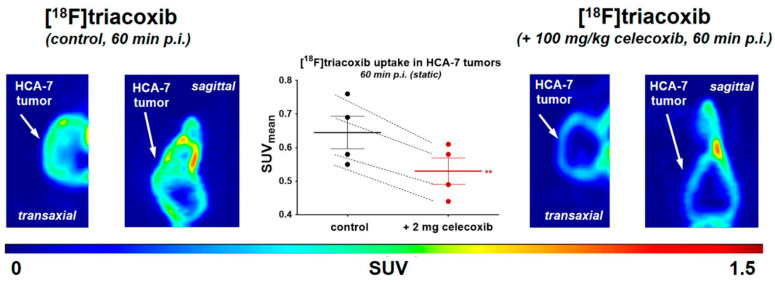
Transaxial and sagittal PET projection of PET images of [^18^F]triacoxib at 60 min p.i. in the same mouse after two consecutive days under control (left) and blocking (right) conditions. Statistical analysis of the blocking effect with 2 mg celecoxib at 60 min p.i. (middle). Data are presented as individual points with the mean ± SEM from *n* = 4 dynamic PET experiments. ** *p* < 0.01. Figure readapted with permission from publisher [90].

Our lab described the in situ click chemistry generation of a highly potent and selective COX-2 inhibitor (COX-2 IC_50_ = 90 nM, COX-1 IC_50_ > 100 μM) [89]. The lead compound was advanced for ^18^F-labelling and PET imaging studies [90]. The Cu-mediated late-stage radiofluorination reaction with boronic acid pinacol ester-based precursor afforded [^18^F]triacoxib in radiochemical yields of 72% (decay corrected) within 90 min, and the molar activity exceeded 90 GBq/μmol (Figure 20). During metabolic stability analysis, ∼90% of [^18^F]triacoxib was found intact after 60 min p.i. The radiotracer showed significant uptake in COX-2 overexpressing HCA-7 cells, but blocking studies with nonradioactive triacoxib suggest the occurrence of unidentified, nonspecific cellular uptake of [^18^F]triacoxib in HCA-7 cells. In pre-clinical PET imaging studies (Figure 21), tumor uptake of [^18^F]triacoxib was noticed, and the pre-treatment celecoxib (2 mg) reduced [^18^F]triacoxib tumor uptake by ∼20% at 60 min p.i. (SUV). It was suggested that the remaining ∼80% of radiotracer uptake and retention in HCA-7 tumors is related to the nonspecific binding mechanisms, including lipophilicity, lysosomal trapping, and off-target binding not related to COX-2. Therefore, it was recommended that additional in vivo experiments should be performed to understand the nonspecific binding mechanisms of [^18^F]triacoxib.

**Figure 22:**
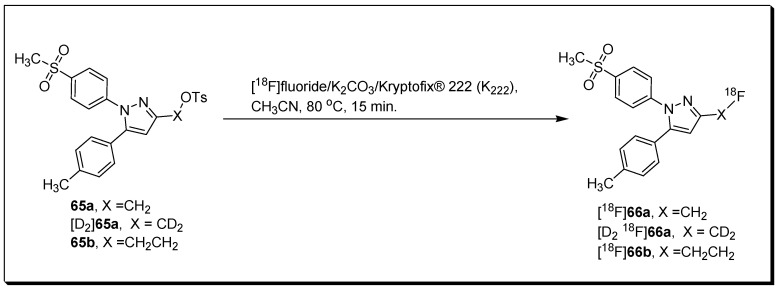
Radiosynthesis of [^18^F]**66a,b** and [D_2_,^18^F]**66a**.

Laube et al. described the radiosynthesis and evolution of three methylsulfonyl-substituted celecoxib derivatives, [^18^F]**66a,b** and [D_2_,^18^F]**66a** [91]. The three radiolabeled compounds were synthesized through a reaction of [^18^F]fluoride with tosylated precursors **65a,b** and [D_2_]**65a** under standard radiolabeling condition in an automated radiosynthesizer (Figure 22). Based on their insufficient COX-2 inhibition potency, the three compounds were not forwarded for in vivo studies in tumor-xenograft-bearing mice. However, in vivo evaluation of biodistribution in healthy mice indicated that the three compounds possess similar pharmacokinetic properties. Upon 5 min p.i., the highest initial radioactivity concentration was observed in liver, adrenals, and brown as well as white adipose tissue. In contrast, dynamic PET studies indicated that [^18^F]**66b** is eliminated more rapidly than [^18^F]**66a** or [D_2_,^18^F]**66a**. An enhancement in metabolic stability was noticed for deuterated compound and a decrease in 18F-defluorination was noticed in the order [^18^F]**66a** > [^18^F]**66b** > [D_2_,^18^F]**66a**.

## 11. 2021

**Figure 23:**
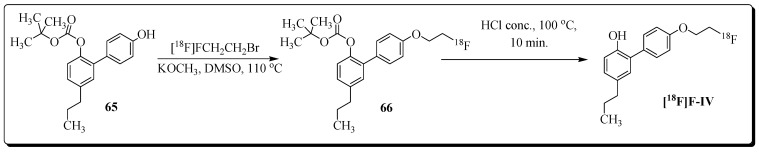
Radiosynthesis of **[^18^F]F-IV**.

The encouraging outcome of a study focused on neuroinflammation imaging with the radioligand [^11^C]MPbP (4’-[^11^C]methoxy-5-propyl-1,10-biphenyl-2-ol) inspired the design and synthesis of a new set of honokiol analogs [92]. All four compounds were tested for their anti-inflammatory activities and compared with celecoxib. Compound (4’-(2-fluoroethoxy)-2-hydroxy-5-propyl-1,10-biphenyl) F-IV (COX-2 IC_50_ = 0.09 µM, SI = 48) was selected for radiolabeling and evaluation in a rat model of LPS-induced neuroinflammation. Radioligand (4’-(2-[^18^F]fluoro-ethoxy)-2-hydroxy-5-propyl-1,10-biphenyl) [^18^F]F-IV was synthesized by radioalkylation reaction of 2-[^18^F]fluoro-1-bromoethane [^18^F]FEB with labelling precursor **65** (Figure 23).

Radioligand **[^18^F]F-IV** was prepared in radiochemical yield of 35% at a molar activity of 35–40 GBq/µmol. Assessment of the ex vivo biodistribution profile of radioligand **[^18^F]F-IV** in LPS-treated and normal Wistar rats showed that the radioligand was able to penetrate the BBB, as high brain uptake was observed with a peak value of 2.21 ± 0.64 and 2.09 ± 0.65 (%ID/g) in the pons and medulla, respectively, at 10 min post-injection. Moreover, blocking experiments with the COX-2-selective inhibitor celecoxib showed a significant reduction of **[^18^F]F-IV** uptake in almost all extracted organs and tissues of LPS rats. The most prominent reduction (20–32%) in radioactivity uptake was observed in the brain (pons and medulla), heart, lung, and kidney. Overall, the outcome of preliminary studies presents **[^18^F]F-IV** as a promising candidate for COX-2-targeting PET imaging of neuroinflammation.

## 12. Conclusions

PET imaging of COX-2 has emerged as an exciting strategy for studying and understanding the role of COX-2 in inflammatory diseases and cancer.

This review summarizes the literature describing the synthesis and evaluation of COX-2-targeting PET radioligands over the last decade. The radiosynthesis of ^18^F-labelled COX-2 radioligands has benefited from recent developments in ^18^F radiochemistry, particularly late-stage radiofluorination. Many ^18^F-labelled COX-2-targeting radioligands with favourable PET imaging characteristics are based on popular selective COX-2 inhibitors like celecoxib. Instalment of ^18^F was either on an aromatic ring or an aliphatic side chain. In addition, recent advancements in [^18^F]CF_3_ radiochemistry should now open new opportunities for designing and preparing COX-2-targeting radioligands at high molar activity using CF_3_-group-containing compounds like celecoxib or mavacoxib. Moreover, complementary imaging techniques like optical imaging have also helped to test novel COX-2 inhibitors and to select promising candidates for radiolabeling with ^18^F.

Despite the challenges associated with high unspecific binding and metabolic stability, the preclinical data of several COX-2-targeting PET imaging agents look promising. The best candidates should be advanced for clinical testing to fully assess the potential and usefulness of COX-2 imaging with PET in the clinic.

## Data Availability

Not applicable.
